# Depression and musculoskeletal diseases: a framework of systemic mechanisms

**DOI:** 10.3389/fpsyt.2026.1977499

**Published:** 2026-10-05

**Authors:** Xiaoyun Yang, Yang Zhao, Wang Gao, Jinkun Liu, Tao Wang, Jingyu Wang, Feixue Wang

**Affiliations:** 1 Department of Traditional Chinese Medicine, Shandong Medical and Pharmaceutical University, Yantai, China; 2 Department of General Education, Yantai Automobile Engineering Professional College, Yantai, China; 3 Department of Transportation Engineering, Yantai Automobile Engineering Professional College, Yantai, China; 4 School of Marxism, Yantai Automobile Engineering Professional College, Yantai, China

**Keywords:** brain-joint axis, depression, gut microbiota, lactylation, musculoskeletal disorders, neuroendocrine

## Abstract

Musculoskeletal disorders (MSDs) encompass a broad spectrum of conditions affecting bones, joints, and muscles, characterized by significant pain and functional impairment, and have become a major global health burden. In recent years, accumulating evidence has revealed complex bidirectional interactions between depression and musculoskeletal health. This review focuses on depression and delineates how it may influence the pathological progression of MSDs through multiple pathways, including neuroendocrine dysregulation, aberrant inflammatory responses, epigenetic modifications, dysregulated extracellular vesicle-mediated communication, abnormal neural activity along the brain–joint axis, and gut microbiota ecological imbalance. Collectively, depression is associated with an increased risk of MSDs and engages intricate interactions across multiple biological systems. Our study underscores that musculoskeletal disorders are by no means confined to isolated joints or local tissues, but rather represent complex diseases with prominent systemic features. Integrated management strategies combining psychosocial interventions, appropriate physical exercise, and dietary modification hold promise as potential multitargeted approaches for the comprehensive treatment of MSDs.

## 1 Introduction

MSDs affect approximately 171 million people worldwide, encompassing conditions such as osteoarthritis, rheumatoid arthritis, fibromyalgia, and sarcopenia (1). These disorders are typically associated with chronic pain, physical functional limitations, disability, and other adverse health outcomes, constituting a substantial global health burden. Emerging evidence suggests that MSDs are not purely biomechanical impairments but are profoundly influenced by psychological factors. Depression is a prevalent comorbidity within this population, and its severity has been correlated with exacerbated pain (2). In addition to elevating annual direct medical costs for MSDs patients (3, 4), depression significantly disrupts daily functioning and diminishes quality of life. Critically, it may also dampen pharmacological responsiveness and curtail the benefits of non-pharmacological therapies (5).

Currently, treatment options for MSDs remain relatively limited, with most existing therapies primarily aimed at symptom alleviation—such as the use of non-steroidal anti-inflammatory drugs for pain reduction (6) or physical therapy to improve joint function—yet these approaches are unable to reverse articular cartilage damage or halt disease progression. Interventions targeting negative emotional states have been shown to reduce pain-related distress (7). For instance, tricyclic antidepressants have been found in small-scale trials to alleviate pain in patients with rheumatoid arthritis (8). Duloxetine has been demonstrated to be an effective and safe therapeutic option for patients with fibromyalgia, irrespective of depression (9, 10). Beyond pharmacotherapy, adjunctive psychological interventions, such as cognitive behavioral therapy, whether employed alone or in combination with exercise therapy, have proven effective in reducing depression and distress (11, 12). Collectively, these findings suggest that depression may represent a novel therapeutic target for musculoskeletal pain. Timely identification and management of depressive symptoms in patients with MSDs are therefore essential for the prevention of degenerative musculoskeletal conditions and for the maintenance of both physical and mental well-being.

Despite the growing recognition that depression plays an important role in the pathogenesis of MSDs, the underlying mechanisms remain insufficiently understood. It remains unclear whether this association arises from response biases induced by repetitive negative thinking, immune-mediated mechanisms affecting inflammation, or the combined influence of psychological, behavioral (13), and biological factors (14) on pain perception. Recent investigations have suggested that the pathological basis may stem from multi-system interactions, including dysregulation of the hypothalamic–pituitary–adrenal (HPA) axis and hormone secretion (15, 16), immune dysfunction (17, 18), dysregulated lactylation modification, aberrant brain-joint axis (19, 20), and gut microbiota dysbiosis (21–23). This complex, cross-system interaction network establishes a vicious cycle between depression and MSDs. Future interventions should effectively address musculoskeletal conditions by integrating psychological support with muscle-strengthening exercise programs (24).

## 2 Information sources and search strategy

This is a comprehensive narrative review that employs a structured literature search. The search was designed to ensure broad and transparent coverage of the relevant human and preclinical literature, with eligibility criteria applied pragmatically to prioritize the most informative studies for each mechanistic pathway. We searched MEDLINE (PubMed), Embase (Ovid), and the Web of Science Core Collection from 1 January 1996 to 10 August 2026 (last updated 10 August 2026, Asia/Beijing). The search combined controlled vocabulary (MeSH/Emtree) with free-text keywords for depression and musculoskeletal outcomes; exemplar terms were (“Depressive Disorder” OR “major depressive disorder” OR depression*) AND (“Osteoporosis” OR “Bone Density” OR “Osteoarthritis” OR “Arthritis, Rheumatoid” OR “Sarcopenia” OR fracture* OR BMD), with additional terms emphasizing immunity, inflammation, nerve activity, and microbiota (“Immunity” OR “Inflammatory factor” OR “Hormone” OR “Brain-joint axis” OR “Microbiota” OR microbiom* OR “short-chain fatty acid*” OR SCFA OR butyrate OR “Probiotics” OR “Exercise”) AND bone-related terms. Inclusion criteria encompassed cohort, case–control, and cross-sectional studies linking depression to common musculoskeletal diseases, randomized controlled trials or quasi-experimental studies with musculoskeletal endpoints, relevant systematic reviews, and preclinical studies (animal models and *in vitro* experiments) that provide mechanistic evidence for the pathways examined in this review. We excluded case reports/series, conference abstracts lacking full data, narrative opinions, and duplicates. Because the review examines both clinical associations and the biological mechanisms that may underlie them, preclinical studies were deliberately retained and are explicitly identified as such in the text. To reduce selection bias, two reviewers independently screened titles/abstracts and full texts, with disagreements resolved by third-party adjudication.

## 3 Neuroendocrine perturbations and their impact on musculoskeletal homeostasis

### 3.1 HPA axis

Depression is closely associated with dysregulation of the HPA axis, which serves as the body’s primary stress response system. Chronic or prolonged stress leads to sustained activation of the HPA axis and persistently elevated cortisol secretion, a critical factor contributing to both depression and bone metabolic disorders (25). In healthy subjects, cortisol has been shown to inhibit renal calcium reabsorption, and persistent HPA axis abnormalities can disrupt calcium homeostasis (26), potentially contributing to osteoporosis and rapid bone loss. *In vitro*, cortisol disrupts bone homeostasis and the synovial fibroblast phenotype in a time-dependent manner: short-term cortisol exposure inhibits osteoblast proliferation, whereas long-term exposure promotes bone resorption by upregulating receptor activator of nuclear factor-κB ligand (RANKL) and downregulating osteoprotegerin (OPG) in osteoblasts (27). These effects also promote bone resorption by enhancing matrix metalloproteinase-13 (MMP-13) activity (28).

### 3.2 Leptin

Beyond its direct effects on bone, depression-induced cortisol elevation also increases leptin levels via stimulation of leptin resistance (29, 30). Leptin, an adipocyte-derived cytokine-like hormone, exerts complex roles in the regulation of joint pain, neuroimmunity, and bone remodeling. It may function as an immunomodulator, activating intracellular JAK2/STAT3, MAPK/ERK, and PI3K/Akt signaling pathways, thereby participating in peripheral metabolism and central nervous system inflammation (31), which in turn contributes to greater pain severity, symptom burden, and disability in patients with osteoarthritis and fibromyalgia (32). *In vitro* studies have shown that leptin acts synergistically with inflammatory cytokines such as interferon-γ (33) and interleukin-1β (IL-1β) (34), increasing the production of inducible nitric oxide synthase, prostaglandin E2, and cyclooxygenase-2 in cartilage (35), inducing chondrocyte apoptosis, and directly participating in the process of joint damage.

### 3.3 Sex hormones

In addition, depression reduces serum levels of testosterone and estrogen (36, 37). Estrogen exerts profound effects on skeletal health by inhibiting osteoclastogenesis and osteoblast apoptosis through the reduction of serum RANKL levels, promoting osteoblast maturation via activation of the Wnt/β-catenin pathway, and increasing OPG levels through upregulation of transforming growth factor-β (38). Consequently, sex hormone imbalance plays a pivotal role in the “neuroendocrine–musculoskeletal” network. In males, it leads to reduced bone mineral density and microstructural deterioration, with a 36% increase in the erosion index and a 16% decrease in bone volume (39, 40). In females, estrogen deficiency disrupts the balance between osteoblastic and osteoclastic activity, reduces bone mineral density, elevates the risk of stress fractures (41), increases the burden of MSDs in postmenopausal women (42), and exacerbates the development of chronic pain (43). Treatment with estrogen (44) or the selective ER modulator raloxifene (45) improved the expression of bone metabolism-related genes and reduced circulating sclerostin levels in postmenopausal women, thereby exerting bone-protective effects. Human umbilical cord-derived mesenchymal stem cells (hUC-MSCs) and their derived exosomes (MSC-Exos) have also been shown to modulate sex hormone homeostasis in male mice, improve the muscle microenvironment, and delay the progression of age-related sarcopenia symptoms (46).

### 3.4 Prolactin

Furthermore, sex hormone deficiency, in conjunction with HPA axis dysregulation, mediates elevated prolactin levels (47–49). Animal model studies have found that prolactin acts through its receptor (50), upregulates the secretion of inflammatory cytokines—such as tumor necrosis factor-α (TNF-α) and interleukin-1—in osteoblasts, increases the RANKL/OPG ratio, and promotes osteoclast formation and bone resorption (51, 52). Conversely, prolactin also inhibits RANKL secretion from synovial fibroblasts, thereby suppressing the formation of mature osteoclasts (53). Collectively, hormones are directly involved in the onset and progression of depression via central mechanisms, while simultaneously modulating bone homeostasis through both direct effects on bone cells and indirect immunoregulatory pathways.

### 3.5 Sympathetic nervous system

Osteoblasts and osteoclasts possess receptors for neurotransmitters such as norepinephrine and neuropeptides (54), suggesting that the sympathetic nervous system may exert a direct role in bone homeostasis. Sustained depression leads to marked and prolonged overactivation of the sympathetic nervous system, resulting in increased release of norepinephrine (55) and neuropeptide Y (56). Aberrantly secreted neurotransmitters may mediate the relationship between depression and bone loss by suppressing osteoblast activity (57), leading to reduced bone mineral density in the lumbar spine and femur, accompanied by decreased trabecular microarchitecture in mice (20). The β-adrenergic receptor blocker propranolol has been shown to improve bone mineral density in osteoporotic mice (58). Additionally, neuropeptide Y receptor antagonists have been shown to alleviate cancer-induced bone pain in rats (59). These findings from animal model studies underscore the role of the sympathetic nervous system in bone metabolism and pain.

In summary, depression-related neuroendocrine abnormalities, including HPA axis hyperactivity, fluctuations in leptin and sex hormone levels, elevated prolactin, and alterations in the sympathetic nervous system, can exert systemic effects on the musculoskeletal system through the neuro-immuno-endocrine network. The reduced anti-inflammatory efficacy of cortisol resulting from HPA axis dysregulation, together with abnormal leptin activation, participates in the metabolic and inflammatory process of rheumatoid arthritis, osteoarthritis, and postmenopausal osteoporosis via central regulation and local adipokine effects. However, these mechanisms differ across different MSDs. For instance, the immunostimulatory effect of prolactin is primarily relevant to rheumatoid arthritis, which is characterized by B cell activation and autoantibody production (60), whereas causal evidence is lacking in osteoarthritis, which is centered on cartilage degeneration (61). β2-adrenergic signaling promotes joint destruction in rheumatoid arthritis and is also involved in pain regulation in osteoarthritis (62).

## 4 Chronic inflammation and immune dysregulation: drivers of tissue degeneration in MSDs

### 4.1 Immune cells and cytokines

The interconnected nature of depression and musculoskeletal health is rooted in shared immune pathways. Depression can induce systemic activation of the immune system (17, 63). Clinical studies have reported that patients with depression exhibit increased neutrophil and monocyte counts, altered heterogeneity of lymphocytes (T cells and B cells), and reduced natural killer (NK) cell cytotoxicity (64). Neutrophils release OPG and express RANK-L and RANK, participating in bone remodeling in patients with chronic inflammatory arthritis such as rheumatoid arthritis (65). Mouse and *in vitro* experiments have confirmed that, within the osteoimmune microenvironment, altered T-cell heterogeneity and reduced NK-cell cytotoxic activity lead to insufficient interferon-γ (IFN-γ) secretion (66). IFN-γ exerts osteoprotective effects by inducing TRAF6 degradation to inhibit RANKL-mediated osteoclast differentiation (67). Macrophage polarization (68) and elevated B-cell numbers (69) increase the release of pro-inflammatory cytokines, including interleukin-17, TNF-α, and interleukin-6 (IL-6). The elevation of these pro-inflammatory cytokine levels upregulates RANKL expression on the surface of bone cells, directly stimulating osteoclast differentiation and activity (70), and disrupting bone remodeling homeostasis through pathways such as PI3K/Akt2, AKT/β-catenin (71, 72) and NF-κB (73, 74) (**Table 1**). Furthermore, inflammatory cytokines such as IL-6 entering the joint cavity can promote matrix degradation, enhance pain sensitivity in patients with osteoarthritis (75), and mediate cartilage degeneration in osteoarthritis. Depression-related immune abnormalities collectively promote osteoclastogenesis, impair the mineralization capacity of osteoblasts, and ultimately lead to bone loss and reduced bone mineral density.

**Table 1:** Inflammatory factors and musculoskeletal diseases.

Pathway	Mechanism	Outcomes	Diseases/species
IL-6	SHP2/MEK2/ERK, SHP2/PI3K/Akt2, AKT/β-catenin, Wnt pathway; reduction of the histone H3K27me3 repressive.	Inhibit the differentiation of osteoblasts, promote bone resorption; inhibit muscle regeneration; aggravate joint inflammation.	Osteoarthritis/human, mice (72); rheumatoid arthritis/human, mice (71); sarcopenia/mice (161).
IL-1β, TNF-α	BDNF release; activation NF-κB pathway, RANKL/OPG imbalance, MMP-9 and MMP-13 increase.	Inducing osteoblast apoptosis, degradation of bone matrix, central sensitization, muscle atrophy	Muscle pain/mice (162); intervertebral disk degeneration/mice (74); rheumatoid arthritis/mice (73); Osteoarthritis/mice (163).
IL-12	Drive T cell and NK cell mediated immune response.	Aggravate inflammation.	Osteoarthritis and rheumatoid arthritis/human (164).
IL-17	RANKL/OPG imbalance.	Bone loss.	Rheumatoid arthritis/mice (70).

### 4.2 Oxidative stress

Oxidative stress is recognized as a core pathophysiological mechanism underlying depression (76) and exerts profound effects on bone metabolic homeostasis through a multi-step cascade. Studies have shown that reactive oxygen species (ROS) play an important role in RANKL-mediated osteoclastogenesis. RANKL, in turn, promotes ROS production in RAW264.7 pre-osteoclast-like cells in a dose-dependent manner, establishing a vicious cycle (77). Inhibiting the endogenous production of ROS and reducing the nuclear translocation of nuclear factor erythroid 2-related factor 2 (Nrf2) in osteoblasts can exert bone-protective effects (78, 79). Curculigoside stimulates the expression of c-Fos and nuclear factor of activated T cells 1 (NFATc1) through activation of Nrf2, and inhibits the NF-κB pathway to alleviate oxidative stress and osteoclastogenesis, thereby regulating bone metabolism in rat models and osteoblasts (80).

### 4.3 Immunotherapy

Existing preclinical studies have explored the potential therapeutic efficacy of targeting the immune system in MSDs. For instance, blockade of IL-6 signaling with tocilizumab has been shown to significantly inhibit microglial activation in the spinal dorsal horn of osteoarthritic rats and alleviate mechanical allodynia (81). Anti-TNF-α therapy with etanercept not only ameliorated anhedonia-like behavior in mice but also attenuated joint inflammation (82). Depletion of neutrophils (83) and CD4^+^ T cells (84) in mouse models has been found to reduce heat hyperalgesia and tactile allodynia. Carnosic acid or irisin can prevent bone loss by promoting Nrf2 nuclear translocation while concurrently inhibiting NF-κB activation (85), and by blocking TRAF6 recruitment to suppress osteoclastogenesis (86). Collectively, the available data not only elucidate the role of depression-mediated immune-related pathophysiology in MSDs but also suggest that this pathway represents a promising therapeutic direction.

Inflammation and MSDs exhibit a shared-skeleton relationship. Inflammatory signaling and immune dysregulation can drive an imbalance between tissue degradation and repair in bone, cartilage, and skeletal muscle. These processes are supported to varying degrees by evidence in rheumatoid arthritis, osteoarthritis, ankylosing spondylitis, and age-related osteoporosis, constituting the pathological basis of the category of inflammatory musculoskeletal diseases. However, the types of triggers that initiate inflammation, the dominant reactive species involved, and the response patterns of target cells differ across diseases. Rheumatoid arthritis is characterized by excessive free radical production and persistent ROS damage (87). Osteoarthritis, by contrast, is dominated by nitrosative stress (88), with synovial lipid peroxidation levels higher than those in rheumatoid arthritis (89). Therefore, future studies should explicitly specify the triggers, dominant reactive species, and target tissues when reporting inflammatory pathways, so as to distinguish disease-specific inflammatory subtypes.

## 5 Lactate as a metabolic–epigenetic nexus: reframing musculoskeletal biology

Depression-mediated tissue inflammation creates a hypoxic microenvironment, which shifts cellular metabolism toward anaerobic glycolysis for rapid energy production. Studies have shown that the mRNA levels of lactate dehydrogenase A (LDHA) and glucose transporter 1 in peripheral blood leukocytes of patients with major depressive disorder (90), as well as lactate concentrations in cerebrospinal fluid (91), are significantly elevated, reflecting systemic metabolic alterations. Studies have confirmed that pathological lactate flux can promote disease-specific mechanisms: in rheumatoid arthritis, elevated lactate in the synovium of patients can promote fibroblast proliferation, thereby driving pathological synovial hyperplasia (92); in a mouse model of obesity-related bone disease, it inhibits lipolysis and osteoclastogenesis in bone marrow-derived macrophages by limiting energy regeneration via lysosomal ATP6V0d2 (93); and in rats with osteoarthritis, it exacerbates catabolic and pro-inflammatory responses in chondrocytes (94).

Beyond its role in energy supply, lactate also modulates gene activity through lactylation, a post-translational modification. Upon entering myogenic cells, lactate induces histone lactylation— predominantly at the H3K9 site—which in turn promotes the expression of the Neu2 gene. As a positive regulator of myogenic differentiation, increased Neu2 expression drives myoblast fusion into myotubes, thereby facilitating muscle regeneration in mice (95). Furthermore, lactylation at the H3K18a site within the promoter region of triosephosphate isomerase 1, mediated by LDHA, has been implicated in the progression of osteoarthritis. Knockout of this modification was shown to increase Col2a1 expression and decrease MMP13 expression in arthritic mouse cartilage, thus promoting cartilage repair (96). In addition to histone lactylation, UDP-glucose 6-dehydrogenase (UGDH) has been identified as a key lactylated protein in chondrocytes following lactate treatment, where lactylation at the K6 site directly inhibits its enzymatic activity, thereby exacerbating extracellular matrix degradation and chondrocyte apoptosis in osteoarthritis mice (97). Collectively, these findings indicate that aberrant lactylation in musculoskeletal diseases is associated with disruption of the osteogenic–osteoclastic balance, extracellular matrix homeostasis, and regenerative capacity (94). Neuroendocrine abnormalities, inflammation, and lactate metabolic reprogramming together form a “depression–immune–metabolism” positive feedback loop. Neuroendocrine dysregulation drives inflammation and reshapes metabolism, while lactate and its lactylation modifications, in turn, amplify inflammatory gene expression and metabolic abnormalities through epigenetic regulation, thereby establishing a self-sustaining pathological cycle. The design of molecular agents capable of enhancing or inhibiting specific protein lactylation events, thereby modulating key signaling pathways involved in muscle growth, differentiation, and repair, may represent a novel therapeutic strategy for MSDs. However, substantial preclinical evidence is still required before such approaches can be translated into clinical practice.

## 6 The role of extracellular vesicles in osteoclast–osteoblast imbalance and muscle atrophy

Since the discovery of extracellular vesicles (EVs), their role in intercellular communication has garnered considerable attention. These vesicles carry a diverse array of bioactive molecules, including proteins and microRNAs (miRNAs), which play critical roles in depression and in the regeneration and pathogenesis of the musculoskeletal system, particularly affecting muscle, cartilage, and joint tissues affected by degeneration. In a rat model of chronic unpredictable mild stress (CUMS), elevated levels of EV-encapsulated miR-126a-3p were detected in serum (92). This miRNA has been shown to inhibit osteogenic differentiation of human adipose-derived mesenchymal stem cells by binding to the 3’-untranslated region (3’-UTR) and suppressing low-density lipoprotein receptor-related protein 6 (LRP6), thereby blocking Wnt signaling activation (98). Similarly, increased serum levels of miR-128-3p in CUMS rats suppress the osteogenic capacity of bone marrow-derived mesenchymal stem cells through targeted inhibition of Smad5 (99). Conversely, certain miRNAs downregulated in serum EVs derived from CUMS rats are also associated with bone remodeling. For instance, miR-455-3p modulates osteoblast proliferation, apoptosis, and oxidative stress via regulation of the HDAC2–Nrf2/ARE signaling pathway, thereby promoting osteoblast growth (100). Unlike endocrine hormones and immune factors, EVs facilitate intercellular communication, modulate immune responses, and promote tissue regeneration through flexible, localized signal delivery, making them promising candidates for future clinical applications in the management of musculoskeletal health and disease. Several ongoing clinical trials offer hope for the treatment of MSDs (101). Nevertheless, the translation of these research findings into clinical practice remains hampered by several challenges, including issues related to large-scale production and isolation, long-term storage and stability, and the development of effective strategies for tissue-specific targeting and delivery.

## 7 The brain-joint axis: neural circuitry linking depression to peripheral joint pathology

The brain–joint axis relies on interactions between peripheral and central signals. Direct neural projections exist between the joints and the brain, forming a bidirectional communication network through neural, endocrine, and immune pathways (102). Depression induces central neuroinflammation, accompanied by elevated levels of IL-6, TNF-α, and C-reactive protein in the brain (103). Transcriptomic studies have revealed that the upregulation of inflammatory cytokine gene expression in the anterior cingulate cortex sensitizes nociceptive conduction circuits in the spinal dorsal horn and thalamus in rats, thereby participating in pathological pain (104). Animal experiments have shown that excessive inflammatory cytokines lead to aberrant neuronal activity in brain regions such as the medial prefrontal cortex, amygdala, and nucleus accumbens, disrupting glutamate (105) and GABA signaling balance (106), as well as dopaminergic signal transduction (107, 108), thereby amplifying pain and emotional signals. Glutamate acts on neural and joint tissues to induce pain, inflammation, and joint degeneration (105). The glutamate receptor antagonist NBQX significantly reduces joint swelling and degeneration in osteoarthritic mice (109). Basimglurant, a negative allosteric modulator of mGlu5, has also demonstrated potential for mood regulation and pain relief in preclinical trials (110). Dopamine treatment can reverse IL-1β-induced NF-κB activation and JAK2/STAT3 phosphorylation, increase the content of type II collagen and glycosaminoglycans, inhibit cartilage matrix degradation, and ameliorate symptoms in osteoarthritic mice (111). These limbic system structures integrate nociceptive and affective signals, and their aberrant neuronal activity is associated with pain catastrophizing in osteoarthritis patients (112), constituting an independent risk factor for the development of central sensitization in MSDs (113).

Brain stimulation techniques, such as transcranial magnetic stimulation (TMS) and transcranial direct current stimulation (tDCS), induce electrical currents in the cerebral cortex, which can either excite or inhibit neuronal activity. Activation of cortical neuronal activity by TMS in patients with knee osteoarthritis has been significantly correlated with pain relief (114). Monthly repetitive TMS applied to the contralateral motor cortex has been shown to ameliorate pain and central sensitization in knee osteoarthritis (115). tDCS combined with conventional rehabilitation may exert potential analgesic effects in patients with fibromyalgia and knee osteoarthritis (116). This effect may be mediated by upregulating N-methyl-D-aspartate receptor expression and inhibiting the BDNF/TrkB signaling pathway in the descending pain modulatory system, thereby alleviating pain in osteoarthritic rats (117, 118). Although TMS and tDCS have demonstrated potential for central neuromodulation in MSDs, their clinical translation remains constrained by multiple limitations: therapeutic efficacy shows considerable disease heterogeneity, with the effectiveness of tDCS in chronic low back pain and myofascial pain syndrome remaining unclear (117); and TMS stimulation parameters lack standardized consensus, making direct comparison across studies difficult. Therefore, they may serve as adjunctive therapeutic options rather than universally applicable routine interventions.

## 8 Gut microbiota as a mediator of depression-induced musculoskeletal pathology: from dysbiosis to systemic inflammation

Depression is closely associated with MSDs and metabolic disturbances, in which gut microbial ecological dysbiosis following stress exposure plays a pivotal role in modulating the progression of musculoskeletal diseases. Clinical studies have demonstrated that Glucagon-Like Peptide-1 (GLP-1), secreted by intestinal L cells, is significantly reduced in patients with depression (119). In GLP-1 receptor knockout mouse models, thyroid calcitonin levels are decreased and osteoclast-induced bone resorption is enhanced, leading to deterioration of normal bone microarchitecture and increased bone fragility. Notably, these effects can be reversed by exogenous GLP-1 receptor agonists (120). In rodents, the GLP-1 receptor agonist liraglutide protects bone by increasing β-catenin and cAMP levels (121–123), while also reducing bone loss and suppressing osteoclastogenesis through downregulation of Trem2, NFATc1, CTSK, and TRAP expression (124). Furthermore, liraglutide targets C2C12 myoblasts to promote muscle formation and function (125). Clinical studies have shown that liraglutide preserves muscle mass in patients with type 2 diabetes to prevent sarcopenia (126) and significantly reduces fracture risk (127).

Depression-related dysbiosis is characterized by a reduction in short-chain fatty acid (SCFA) producing taxa and a decrease in gut microbial alpha diversity. SCFAs such as butyrate play important roles in Treg cell differentiation (128) and in the regulation of inflammation (129, 130). When butyrate levels are diminished, the activation of NF-κB and NLRP3 may become more pronounced, promoting the accumulation of pro-inflammatory mediators such as IL-1β (131, 132). Sustained butyrate release at the colonic site using nanoparticles can modulate gut microbiota composition and downregulate pro-inflammatory responses, thereby alleviating bone loss in mouse models (133). Similarly, multi-strain active probiotics can reshape the gut microenvironment in rats with osteoarthritis and reduce systemic or joint inflammatory markers (134). A Mediterranean diet rich in monounsaturated fatty acids, ω-3 polyunsaturated fatty acids, and dietary fiber has positive effects on reducing pain and improving physical function in patients with rheumatoid arthritis (135); low-energy diets such as low-carbohydrate diets have shown potential in alleviating pain and improving physical function in patients with osteoarthritis (136); and the therapeutic potential of probiotic supplementation in improving bone metabolism and pain in patients with movement disorders has also been recognized (137). Current evidence supports their role in relieving pain, improving musculoskeletal function, and exerting anti-inflammatory effects, offering a practical dietary pattern for patients with musculoskeletal diseases.

Furthermore, appropriate physical exercise (e.g. aerobic, resistance, and combined training) not only ameliorates emotional disturbances and pain in patients with MSDs (138), but also yields sustained improvements in muscle performance and skeletal function (139–150) (**Table 2**). A single bout of high-intensity exercise has been shown to increase circulating extracellular vesicles enriched in redox-regulating enzymes, which influence vascular structure and function in mouse skeletal muscle in a manner dependent on the antioxidant enzyme glutathione peroxidase 1 (151). Regular moderate-intensity exercise is associated with higher bone mineral density at the lumbar spine and femoral neck, and contributes to a reduced risk of fractures (152). Sustained physical activity also supports gut microbial diversity and SCFA-related metabolism (153), enhances metabolic homeostasis, reshapes body composition (154), suppresses inflammatory responses (155), and preserves muscle function while mitigating muscle atrophy (156). As an effective bridge between psychological intervention and healthy lifestyle management, integrated strategies combining exercise, dietary modulation, and targeted psychological support may yield superior outcomes in pain relief and tissue functional recovery in patients with musculoskeletal conditions. The gut microbiota links environmental factors (such as stress, diet, and exercise) to systemic immune and metabolic status, acting simultaneously on the immune, metabolic, and neuroendocrine systems, thereby providing a practical entry point for lifestyle-based interventions.

**Table 2:** Neuroimmune pathways and musculoskeletal diseases.

Musculoskeletal diseases	Exercise strategy
Knee osteoarthritis	Yoga or strengthening exercise (twice weekly for 24 weeks) (141); aerobic exercise (4 and 12weeks) (142); personalised gait retraining (5° or 10°change in foot progression angle) (143).
Rheumatoid arthritis	High intensity interval training and strength exercise (12weeks) (144, 145); aerobic exercise (twice weekly for 6 weeks) (146).
Fibromyalgia	CBT combined with neuromuscular exercise (twice weekly for 8 weeks, with each session lasting 75 to 90 minutes) (147); lifestyle physical activity (five to seven days per week, once for 30minutes) (148).
Sarcopenia	Progressive resistance exercise (12 and 24 weeks) (149, 150); high intensity interval training (139)

## 9 Conclusion

The relationship between depression and MSDs has evolved from descriptive associations into a systematic theoretical framework with increasingly well-defined pathogenic mechanisms. This review delineates how depression may contribute to the development of MSDs at multiple levels, encompassing neuroendocrine dysregulation, immune activation, lactylation modification, an aberrant brain-joint axis, and gut microbiota imbalance (**Figure 1**). Collectively, these interdependent processes constitute a multifactorial network that offers novel perspectives for understanding musculoskeletal disorders as complex inflammatory and degenerative diseases. Furthermore, this framework highlights the regulatory mechanisms spanning from local articular structures to systemic homeostasis, thereby providing a theoretical foundation for the development of therapeutic strategies in MSDs.

**Figure 1:**
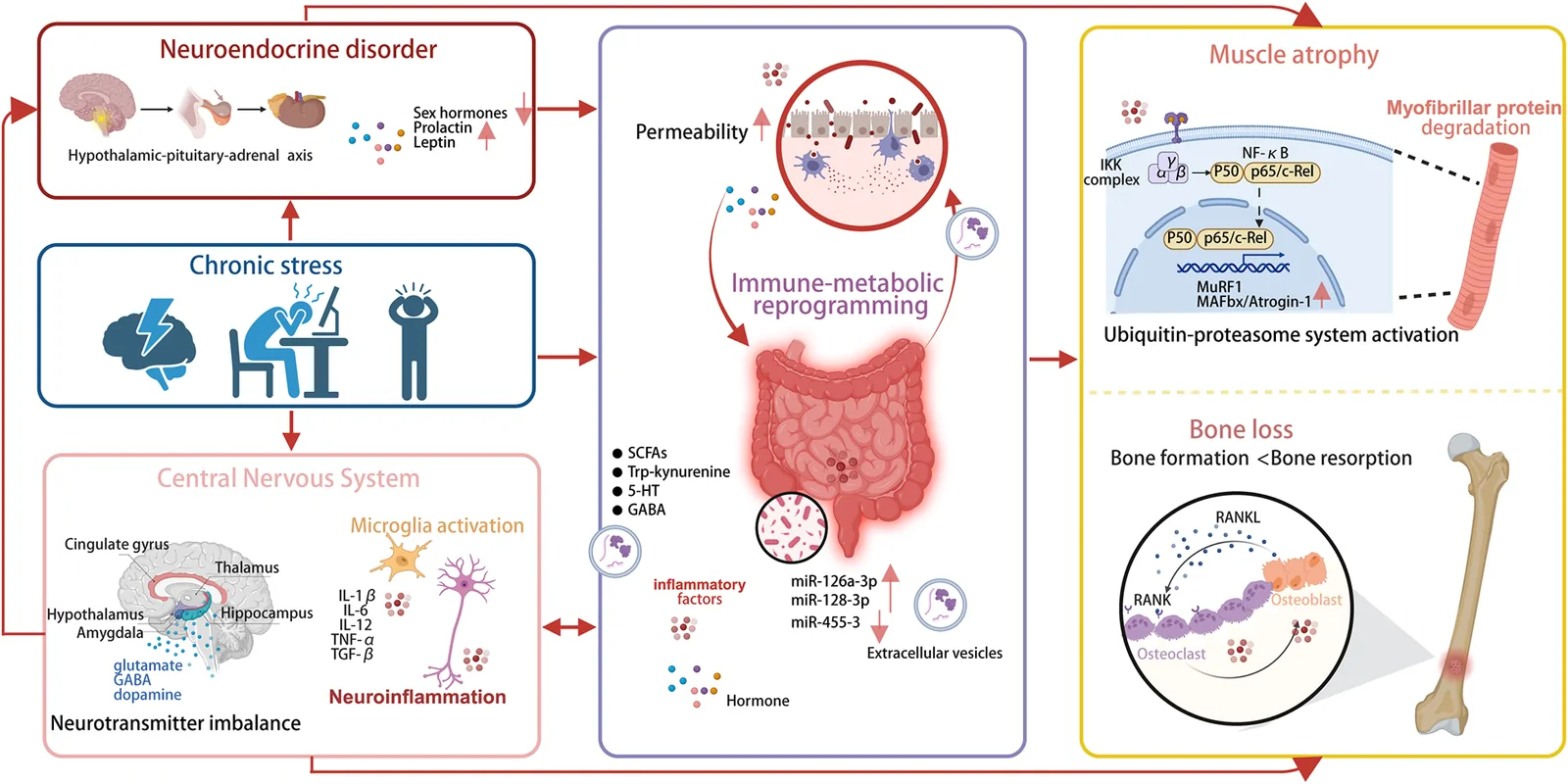
Schematic diagram of proposed pathological mechanisms linking depression to musculoskeletal diseases. Musculoskeletal tissues are subject to modulation by depression, resulting in muscle atrophy, joint inflammation, and bone loss. Depression is associated with HPA axis hyperactivity and abnormal neuronal firing in limbic circuits, alongside gut microbiota dysbiosis and systemic inflammation. Such multisystem crosstalk further accelerates the pathogenesis and progression of musculoskeletal diseases.

The above sections describe distinct biological systems. However, these systems do not operate in parallel or independently, but rather constitute an interconnected network. Existing evidence suggests that this process may follow a temporal sequence: it begins with depression-related chronic stress and neuroendocrine activation, followed by systemic immune and metabolic reprogramming, then tissue-level degeneration, and ultimately central sensitization and pain amplification, after which the brain–joint axis and associated behavioral changes sustain this cycle. This network comprises multiple closely interconnected components, including the HPA–immune axis, the inflammation–lactate–lactylation pathway, the brain–joint pain–limbic neurocircuit, and the gut–immune axis. This also explains why single-target interventions for MSDs have limited efficacy and why multi-system coordinated treatment is required. However, direct longitudinal evidence for this network in humans is currently lacking; therefore, this framework should be regarded as a working model rather than an established sequence.

In the existing literature, most human evidence exploring the association between depression and musculoskeletal disorders comes from cross-sectional surveys (2, 13) or retrospective cohorts (3, 4). Cross-sectional designs cannot distinguish the temporal sequence of “depression leading to musculoskeletal pathology” versus “musculoskeletal pathology leading to depression”, nor can they exclude the possibility of shared third variables. Prospective cohort studies and interventional studies are needed to verify causality. Moreover, much of the evidence synthesized in this review derives from preclinical studies and *in vitro* experiments. Although these models reproduce some features of human disease, they cannot fully capture the complexity, chronicity, and comorbid burden of clinical depression and MSDs, and species- and time-dependent effects may not directly translate to patients. Definitions and measurement methods for depression vary substantially across studies, ranging from clinician-diagnosed major depressive disorder to self-reported depressive symptoms and stress indices, with these measures differing in severity thresholds and assessment instruments, thereby hindering direct comparison and pooled analysis. Finally, although the molecular targets discussed are promising, they remain at preclinical or early experimental stages, and their safety, efficacy, dosage, and tissue-specific delivery mechanisms in humans have not yet been clarified (94, 101). Therefore, the clinical implications of this framework require validation through prospective, adequately powered studies that prioritize interventional designs.

Beyond the biological framework, these findings carry important implications for psychiatric assessment and public mental health. For MSDs patients, depressive symptoms should be systematically assessed, including somatic manifestations such as pain, fatigue, and sleep disturbances. Early identification and intervention for depression may reduce postoperative complications after musculoskeletal surgery and improve long-term functional outcomes. It should therefore be incorporated into multimodal management frameworks, with screening for depressive symptoms integrated as part of bone health management. Furthermore, given that depressive symptoms often persist beyond the rehabilitation period (157), longitudinal mental health monitoring should be embedded within chronic disease management pathways to identify patients requiring intensified care. Meta-analysis of psychological interventions have confirmed their significant effectiveness in alleviating pain and functional impairment in MSDs patients (158–160). Intervention approaches should be tailored to the severity of depressive symptoms, and practical, feasible practices should be explored in the musculoskeletal field through collaborative care models. Integrating psychological assessment into musculoskeletal health management represents an important step toward the comprehensive, person-centered care model advocated by both psychiatry and musculoskeletal medicine.
